# The effect of external ankle support on knee and ankle joint loading in netball players

**DOI:** 10.1186/1757-1146-5-S1-O51

**Published:** 2012-04-10

**Authors:** Benedicte Vanwanseele, Max Stuelcken, Andy Greene, Richard Smith

**Affiliations:** 1 Health Innovation and Technology Department, Fontys University of Applied Sciences, Eindhoven, The Netherlands; 2 Exercise, Health and Performance Research Group, The University of Sydney, Australia; 3 Departement of Biomedical Kinesiology, KULeuven, Leuven, Belgium

## Background

External ankle support has been successfully used to prevent ankle sprains [1]. However, some recent studies [2,3] have indicated that reducing ankle range of motion can place larger loads on the knee and increase the risk of knee injuries. The aim of this study is to investigate the effect of external ankle support (braces and high top shoes) on ankle kinematics and knee kinetics in high performance netball players.

## Materials and methods

Eleven high performance netball players were recruited from NSW Institute of Sport. A 14-camera motion analysis system was used to synchronously collect three-dimensional video and force plate data. Twenty-four retro-reflective markers were attached to anatomical landmarks to allow the formation of rearfoot, forefoot, shank, thigh, and pelvis segments. Each player performed a single-leg-landing whilst receiving a chest pass. There were three conditions: a standard netball shoe (Ignite 3, ASICS), a standard netball shoe in conjunction with a semi-rigid ankle brace, and a high top basketball shoe (Jordan, NIKE). Five trials were analysed for each condition. Comparisons between the brace and the standard shoe conditions; and between the high top and the standard shoe conditions were made using the Wilcoxon sign-rank test.

## Results

**Figure 1:**
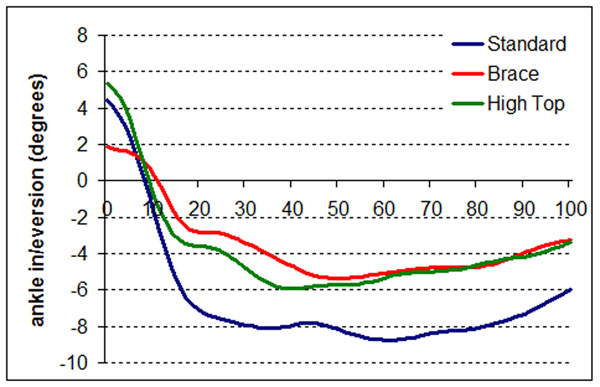
Average time series for the ankle angle in the frontal plane during a single-leg-landing wearing standard netball shoes, standard netball shoes with brace and high top shoes.

The maximal ankle eversion angle was significantly larger in the standard shoe condition compared to the brace condition (12.35±8.62 vs 7.79±4.60 degrees, p=0.038) (Figure 1). The same trend was observed when comparing the standard and high top shoe conditions (8.79±3.87 degrees) but significance was not reached.

None of the moments were significantly different between the conditions but there was a trend for an increased ankle plantarflexion moment and hip flexor moment in the brace condition compared to the standard shoes.

## Conclusions

Although the ankle eversion angle was restricted by use of an external brace, no changes in the knee and ankle joint moments were observed.
